# Notes and News

**Published:** 1893-12-09

**Authors:** 


### Notes and News.

Collected and Communicated.

The General Council of the Seine has formulated a project of establishing a home for inebriates in Paris, in view of curing the same. This proposition is likely to meet with much substantial support.

The inauguration of the International Hospital took place recently in Paris, under the presidency of M. Jules Simon. This establishment possesses incontestable advantages, opening its doors to foreigners as well as to citizens, and to all sick poor without distinction of race or religion.

The White Star Line mail steamer "Gothic" is to be placed on exhibition in aid of the funds of the Seamen's Hospital and its dependencies on Saturday, December 16th. The "Gothic" is the largest ship, with exception of the "Great Eastern," which has ever entered the Port of London. The arrangements of the "Gothic" are most complete and modern, and as the whole of the ship is open to inspection a most interesting opportunity will be afforded the public of viewing one of our most imposing water palaces. Trains run every half-hour from Fenchurch Street to Manor Way Station, which is alongside the vessel in the Royal Albert Dock. The entrance fee of one shilling may be made on board.

The Statistical Report issued by the Metropolitan Asylums Board, which is regarded as a somewhat antiquated document by the critical, nevertheless contains a fact which alone should make it a record of general interest. This fact is that of the 2,000 persons employed by them in their fever hospitals only three died of diseases contracted in the discharge of their duties of ministering to the sick. This result should increase confidence in the management of the fever hospitals, and should also encourage those who, whilst excellent and courageous nurses in other respects, dread the risks attendant on the nursing of small-pox cases more especially. The risks of attendance on infectious diseases is minimised where the general hygienic and sanitary arrangements are good, and, in spite of the difficulties under which the Board labour in respect to their hospitals, we feel we must congratulate them on a condition which can afford so good a record.

Professor Garner is still an enthusiastic scholar of Monkey-lore. His close study of monkeys and their ways has resulted in the opinion that they are guided by definite sounds, rather than by gestures—as has been supposed. He has manufactured a variety of instruments reproducing various sounds, which his investigations so far have enabled him to identify with actual intention. In the *Pall Mall Budget* for November 30th these instruments are reproduced. Their scope appears to be so limited that an equal variety might be produced from the most cursory study of bird calls.

Anti-vivisectionists have found a scope for their energies in reference to the Bill now under consideration of the Indian Legislature as to the legalisation of vivisection experiments, hitherto prohibited in India. A protest has been signed and forwarded to Lord Landsowne. If vivisection is to be permitted at all, however, it is urged that the strongest restrictions and regulations should be enforced. The Society for the Protection of Animals from Vivisection have gone further than this, inasmuch as they oppose the establishment of a Pasteur Institute in India. They maintain that the results of the Pasteur treatment do not justify the sufferings entailed on animals in whom the malady has to be perpetuated for curative purposes in human beings.

The quarterly court of the Hospital for Consumption, Brompton, was held last week. Mr. T. B. Beckwith presided. In commenting upon the report of the Board of Management, read by Mr. Theobald, the new secretary, he said that the most important event in their recent history had been the appointment of Miss Charlotte Davidson, from Newport, to succeed Mrs. Elliott as lady superintendent; they had every reason to believe that a good choice had been made out of the eighty-one applicants. Their income from legacies had fallen off. In the fact that they had 177 applicants waiting for admission they had proof that, despite the progress that had been made in medical science, there was still as great a need for their hospital as ever. It was reported that Dr. H. Stanley Ballance had been elected resident medical officer and Dr. J. J. Perkins assistant medical officer. Mrs. Gand and Mr. Richard Dawe were elected honorary governors. The meeting closed with a vote of thanks to the chairman, moved by Mr. R. B. Chapman.

At the quarterly court of the governors of Middlesex Hospital, held last week, Mr. H. N. Custance presiding, it was intimated that it would be necessary to sell stock to the amount of £9,000. As with most hospitals, the revenue, particularly from legacies, has fallen off greatly, and Middlesex is in debt to the sum of £8,000. In the report of the board of managers, reference was made to the deaths of Mr. Freer Lucas, casualty medical officer, who contracted diphtheria while assisting at an operation upon a diphtheritic patient; and of Mr. Arthur Hensman, aural surgeon to the hospital. Negotiations for the purchase of a site for a convalescent home at Clacton-on-Sea have been concluded. Sir Ralph Thomson, K.C.B., and Rev. Canon Acheson, have been appointed members of the board, in room of Lord Muncaster and Rev. Hay Chapman, resigned. The report mentioned that Middlesex Hospital has been fortunate in regard to the coal strike, inasmuch that just before the strike began a large quantity of coal was purchased at the contract price of 17s. 6d. per ton.

